# Submucous fibrosis of the oral cavity. Histomorphological studies.

**DOI:** 10.1038/bjc.1966.79

**Published:** 1966-12

**Authors:** P. N. Wahi, U. K. Luthra, V. L. Kapur

## Abstract

**Images:**


					
676

SUBMUCOUS FIBROSIS OF THE ORAL CAVITY

HISTOMORPHOLOGICAL STUDIES

P. N. WAHI, USHA K. LUTHRA AND V. L. KAPUR

From the Department of Pathology, S.N. Medical College, Agra, India

Received for publication May 27, 1966

CLINICAL, epidemiological and cytomorphological studies in cases of sub-
mucous fibrosis have been the subject of separate communications (Wahi et at.,
1966a, b, c, unpublished). The majority of the studies (Sharan, 1959; Rao,
1962; Sirsat and Khanolkar, 1957, 1962) have described the histological changes
of the subepithelial tissue in submucous fibrosis. Some have more comprehensively
dealt with the histochemical and electron microscopic studies of this condition
(Sirsat and Khanolkar, 1957, 1960, 1962). Only an occasional report in the
literature (Pindborg et al., 1965) describes the epithelial changes in detail in cases
of submucous fibrosis.

None of the studies has dealt with the histological changes in cases of submucous
fibrosis relating them to the severity and the extent of involvement of the leison,
i.e. according to the clinical groups of these cases (Wahi et al., 1966a, unpublished).
Factors concerned in the aetiogenesis of submucous fibrosis are not clearly
understood. The use of tobacco, with associated mechnical or thermal trauma,
acting in conjunction with vitamin deficiencies, is considered to play an important
role in the development of the lesion (Wahi et al., 1966b, unpublished). The
present paper deals with the detailed histomorphological features in 104 cases of
submucous fibrosis in relation to the severity and the extent of involvement of
the lesion, and according to the tobacco consumption habits.

MATERIAL AND METHODS

A total of 104 punch biopsies were taken under local anaesthesia. According
to the site of involvement 53 biopsies were taken from the palate, 46 from the
buccal mucosa, 3 from the tongue, and 1 each from gingivae and lip.

All biopsies were fixed in 10 per cent formol saline, embedded in paraffin and
cut in serial sections. Sections were stained with haematoxylin and eosin, a
modified Mallory's connective tissue stain (Weinmann and Meyer, 1959), periodic
acid Schiff reagent and toluidine blue stain. Sections were evaluated in detail
for changes in the epithelium, juxta-epidermal region and the corium.

OBSERVATIONS

Detailed histomorphological studies are presented in 104 cases of submucous
fibrosis. Histochemical studies were carried out in some of these cases. Histo-
morphological changes in cases of submucous fibrosis have been dealt under the
following headings:

(1) Changes in epithelium

(2) Changes in subepithelial tissue

SUBMUCOUS FIBROSIS OF THE ORAL CAVITY

Changes in epithelium (see Table I).

The majority of cases show epithelial hyperplasia (Fig. 1), though rarely one
may find normal or atrophic epithelium (Fig. 2). Of the 31 cases of submucous
fibrosis in clinical group I, 28 cases (90.4 per cent) show hyperplasia, 2 cases
(6.4 per cent) atypical epithelial hyperplasia (Fig. 3 and 4) and 1 case (3-2 per cent)
atrophic changes. Somewhat similar observations are seen in 60 cases of clinical
group II. Here 55 cases (91F6 per cent) show hyperplasia, 4 cases (6-6 per cent)
atypical epithelial hyperplasia and 1 case (1.6 per cent) a normal epithelium.
However, striking differences in the epithelial changes are observed in group III
cases; of the 13 cases encountered the epithelium shows atypical epithelial
hyperplasia in 9 cases (69.2 per cent) and the remaining 4 cases (30.7 per cent)
show hyperplasia. Four cases of submucous hyperplasia were associated with
epidermoid carcinoma; in the areas of submucous fibrosis the epithelium shows
atypical epithelial hyperplasia in all these cases.

Interesting observations are discerned on studying the epithelial changes in
relation to the tobacco and betal chewing habit of the patients (see Table II).
Of the 22 cases with no habit, 20 cases (91-0 per cent) show epithelial hyperplasia
and the other 2 show normal or atrophic epithelium. In none of these cases does
the epithelium show atypical epithelial hyperplasia. Amongst the 6 cases using
betal nuts and betal leaf, 5 cases (83-4 per cent) show epithelial hyperplasia and
1 case atypical epithelial hyperplasia. On the other hand patients using tobacco,
as cigarette smoking or tobacco chewing or both habits, showed a gradual decrease
in the number of cases showing epithelial hyperplasia, and an apparent increase
in the number of cases showing atypical epithelial hyperplasia (Table II). All
the 4 cases of submucous fibrosis associated with epidermoid carcinoma were
tobacco users, 3 had the combined habit of chewing and smoking, and one only
chewed.

Considering the epithelial changes in the 28 non-tobacco users, 25 cases (89.2
per cent) show epithelial hyperplasia, 1 case (3-6 per cent) atypical epithelial
hyperplasia and 1 case (3-6 per cent) each of atrophic and normal epithelium.
However, of the total 76 cases using tobacco in one form or the other, 62 cases
(81.6 per cent) show epithelial hyperplasia and 14 cases (18.4 per cent) atypical
hyperplasia (Table III). Atypical epithelial hyperplasia is thus an important
feature in cases of submucous fibrosis using tobacco as compared to the cases
which do not use tobacco.

The squamous epithelium in all the cases of submucous fibrosis shows kerati-
nizing metaplasia-orthokeratosis (Fig. 5) (40 cases), parakeratosis (Fig. 6)
(45 cases), or both ortho- or parakeratosis (19 cases). No distinct difference is
observed in the type of keratinization in the various clinical groups of cases of
submucous fibrosis.

However, interesting observations are discerned when the type of keratiniza-
tion is correlated with the site of the lesion and the habit of the patients. The two
commonest sites involved by submucous fibrosis are the palate and the buccal
mucosa (Wahi et al., 1966a, unpublished). The palate is predominantly involved
in 53 cases, and buccal mucosa in 46 cases. Of the 53 cases with palatal involve-
ment, 22 cases (41.5 per cent) show orthokeratosis with or without hyperplasia,
11 cases (20.7 per cent) parakeratosis with or without hyperplasia, and the
remaining cases a combination of these two types of keratinization. Of the 46
cases involving buccal mucosa, 20 cases (45-2 per cent) show parakeratosis with

677

P. N. WAHI, U. K. LUTHRA AND V. L. KAPUR

or without hyperplasia, 10 cases (21.7 per cent) orthokeratosis with or without
hyperplasia, and the remaining cases a combination of these two types of
keratinization.

It is thus seen that lesions of palate predominantly showed orthokeratosis,
and those of buccal mucosa parakeratosis. The frequent clinical recognition of
lesions involving the palate (Wahi et al., 1966a, unpublished) may be aided to some
extent by the nature of keratinization involving the different sites of lesion. Of
the 4 cases of submucous fibrosis associated with epidermoid carcinoma the
epithelium  was parakeratotic in 3, and showed a combination of parakeratotic
and orthokeratotic epithelium in the remaining one.

The type of keratinization according to habit in cases of submucous fibrosis
is shown in Tables II and III. Orthokeratotic epithelium predominates in non-
tobacco users, i.e. 17 cases (60-8 per cent) and parakeratotic epithelium in tobacco
users, i.e. 39 cases (51.4 per cent).

The rete pegs are more frequently pointed as the cases advance from group I
to group III (41.9 per cent to 77-7 per cent) (Table I, Fig. 7). Moreover, pointed
rete pegs are also more frequently seen in tobacco users (72.3 per cent) than in
non-tobacco users (46.4 per cent) (Table III). Stratum granulosum is either
distinctly visualized or is patchy or completely absent; it is more frequently
patchy or absent in tobacco users than in non-tobacco users (Table III).

Spongiosis is more frequently observed in cases using tobacco, and compara-
tively less frequently in non-tobacco users (Tables II and III).

The basement membrane is discernible in the majority of the group I and
group II cases, whereas it is focally indistinct in a fair number of group III cases
(Table I). Interestingly the basement membrane is distinctly discernible in all
the non-tobacco user cases, whereas it is indistinct in 73-8 per cent of tobacco
users.

The number of mitotic figures were counted per 10 high power fields in cases
belonging to various clinical groups (Table I), and also in cases according to habit
(Table II and III). In group I cases the maximum number of cases, 19 (61.3
per cent), reveal 3-4 mitoses/10 high power field and none shows figures above 8
mitoses per 10 high power field, whereas in group II the maximum number of
cases, 26 (43.3 per cent), show 5 to 6 mitoses per high power field and 2 cases

EXPLANATION OF PLATES
FIG. 1.-Epithelium showing hyperplasia. H. & E. x 85.
FIG. 2.-Epithelium showing atrophy. H. & E. x 360.

FIG. 3.-Epithelium showing atypical epithelial hyperplasia. H. & E. x 85.

FIG. 4.-Epithelium showing atypical epithelial hyperplasia. H. & E. x 360.
FIG. 5.-Epithelium showing orthokeratosis. H. & E. x 85.
FIG. 6.-Epithelium showing parakeratosis. H. & E. x 85.

FIG. 7.-Epithelium showing blunt and pointed rete pegs. H. & E. x 360.

FIG. 8.-Epithelium showing parakeratosis, rete pegs predominantly blunt. Juxta-epithelial

tissue and superficial corium showing dense hyalinization fibrosis and constriction of blood
vessels. H. & E. x85.

FIG. 9.-Juxta-epithelial tissue and superficial corium showing dense and fibrillar connective

tissue. H. & E. x85.

FIG. 10.-Juxta-epithelial tissue and superficial corium showing dense hyalinization, fibrosis

and constriction of blood vessels with slight endothelial cell proliferation. H. & E. x 360.
FIG. l1.-Juxta-epithelial tissue and superficial corium with fibrillar connective tissue showing

dilated and congested blood vessels. An occasional blood vessel shows slight medial
thickening. H. & E. x 360.

678

BRITISH JOURNAL OF CANCER.

1

2

3                          4

Wahi, Luthra and Kapur.

Vol. XX, No. 4.

BRITISH JOURNAL OF CANCER.

6

7                          8

Wahi, Luthra and Kapur.

Vol. XX, No. 4.

F. "

BRITISH JOURNAL OF CANCER.

9                                        10

11

Wahi, Luthra and Kapur.

VOl. XX, NO. 4.

SUBMUCOUS FIBROSIS OF THE ORAL CAVITY

(3 3 per cent) above 8 mitoses per 10 high power field. However, in group III
although the maximum number of 6 cases (46-1 per cent) show 3-4 mitoses per
10 high power field, a considerable number, 3 (23.0 per cent) of the total 13, show
mitoses above 8 per 10 high power field (Table I). In the 4 cases of submucous
fibrosis associated with epidermoid carcinoma the mitotic count was between 6
and 13 per 10 high power field.

Considering the number of mitotic figures according to habit, a higher mitotic
figure count is found in tobacco users than in non-tobacco users. Of the tobacco
users, higher mitotic counts (above 8 per 10 high power field) are more frequently
encountered in patients with the combined chewing and smoking habits.

Changeys in subepithelial tissue

In most of the cases of submucous fibrosis, the juxta-epithelial tissue shows
dense hyalinization and fibrosis (Fig. 8). There is a distinct increase in the
frequency and severity of this observation from cases of clinical group I to group
III. It is observed in 12 cases (38.7 per cent) of group I, 43 cases (71-6 per cent)
of group II and in 12 cases (92.3 per cent) of group III cases. The remaining
cases show combination of the other types of juxta-epithelial tissue-i.e. dense
and fibrillar (Fig. 9), loose and fibrillar and loose and hyalinized. In all the 4
cases of submucous fibrosis associated with epidermoid carcinoma the juxta-
epithelial tissue is dense and hyalinized. According to the habit, dense and
hyalinized type of juxta-epithelial tissue is present in 12 cases, i.e. 92-3 per cent
of cases with the habit of tobacco chewing and smoking, and in 12 cases, i.e. 75-0
per cent of those with the habit of chewing alone (Table IV).

In the majority of cases the blood vessels in juxta-epidermal connective tissue
are constricted (Fig. 10). This observation is seen in increasing frequency from
group I to group III cases (Table V). This feature is also commoner in tobacco
users, i.e. 59 of 76 cases (77.7 per cent) than in non-tobacco users, i.e. 11 of 28
cases (39.2 per cent) (Table VI). In the remaining few cases the blood vessels are
either dilated (Fig. 11) or normal. In all the 4 cases of submucous fibrosis associ-
ated with epidermoid carcinoma, there is marked constriction of the blood vessels
in the juxta-epithelial connective tissue. Inflammation of the juxta-epidermal
connective tissue was a rare feature in cases of submucous fibrosis.

Toluidine blue metachromasia was studied in a total of 58 cases of submucous
fibrosis. The results of the reactions are expressed as negative, slight metachro-
masia +, moderate metachromasia +, and marked metachromasia + +. The
maximum number of group I cases, 10 (45.4 per cent), gave a negative reaction,
whereas the maximum number of group III cases, 4 (50-0 per cent) gave a markedly
positive metochromasia (Table VII). All 4 cases of submucous fibrosis associated
with epidermoid carcinoma gave a markedly positive toluidine blue metachromasia.

Interestingly, 9 cases (90.0 per cent) of submucous fibrosis with no habit, and
all 4 cases using betal nut leaf gave a negative reaction. On the other hand, the
metachromasia increased in severity and frequency from tobacco smokers to
tobacco chewers being maximum in those with the combined habit of chewing and
smoking (Table VIII). Histochemical tissue staining reactions for maturation
of the collagen were carried out in some cases. There is greater evidence of
maturation defect of connective tissue in cases of clinical group III, than in cases
of clinical groups I and II. Moreover, the non-tobacco users did not show any

679

P. N. WAHI, U. K. LUTHRA AND V. L. KAPUR

M.0

I8 AoAqV I c6q

0oM0

9

9 1 - 6   C1

Go
CZ

0

04
c)
Co

P-Q

Co
Co

C4

,a[  *H 1aunsmO p1u  .? .
ouvjqtuauw quaaeng 11o0

aulJquoau wuaiuoseg c* , co

s!soiBuodS A X

10 t 10

luasqu cO
unsolnuuis unuitl  gq c

Aralpd ' C

tunsolnuuSl muiwnzelS 0 D

quasaid '? .

unon unl  1000-

CO1O
0-101003

-adpuv -odlj  A 6 r

ISIJUOdXq -vare

101

'U100-
UIl{go ( O

palulCd  6 A i
Aqdo?lVYC

00CO

vismidiadAq +?t
vislqdjadiSHo  o

CDOCO
OU Q

I.

0.

-._

8 oAoqV        I I      I

C')

la V.]
ou?uqmeuu

eutujqcuou

tunsolnuus
umsolnuue

siso,

CI

an

4)1
4)

L

I

9-1s    0

C0 go  10 c

t-   I-4 1C c

Z,-O  I eC C CO

00 co rCO s

Spa!d 0 -1 co km

J zu1s11J  Oo +  ,-4o
uR tunjIS 01c  -001

pU   -1NX 0  0 ) 1 1

01-4 0COCO

CuaQi _? ?  09 X'7

PJQmIOq1fO C o  o A

so CO01

CO

cqv10 100CO c
t I

A4doJs 41   1 1 I
)IM A)0w  10

!41ldItadXq   C  Oo
To l'0dA 1 CO 00

da  viX mo  o>to

0uni    0-00
pouo so

PsLJOd  N     I I II

o    0

IC

co

0e be be

.  '0

4) 4)0

ZQ -Co Q C) to

03o        n ,

680

la

0i

.;4

;r

Co

P-Q4

*e.0

Co

01.

-'4

I.

-ttb
Dq

o 4

UD F
to

0

4)1

Lg

I

SUBMUCOUS FIBROSIS OF THE ORAL CAVITY

la i I
auvjqxua

tunsolnui
uinolnul
wnsolnui

gqig
11 l14

PUN

I!B

8 gAoqV

toC

El

8-L  l

01

C   1
to to

puu -oqspu!

OIVJ93101ql01
*H  unls   .? cli

o   ci
0 P
visouodS A d

cq
AIplIld 4
L12 u1I1eJ}S A

01

lueseid 9 Ob
BlD wns    S

)slVJIaToq1O 0 0

to to

t 01

t] o

tulgo  01
poluloti to 01
Aqdol}y % I
da lwosAlVX  X
,eI3Id9dAH o

lItUJON X

.2

0b

8)

00
p CD
* )

I.

*H

8 @

44

+ juatum!da
UOTleUIIUU11UI

P@lXIIP to

91069,9A P??lgE |

paSaIiSsuoo o
g1eggsA Poolif -;

l,uuou ~

Sj6OsWA POOIi[ OD g

pGZIrnl,Sq 4 9
pus OBoo'l   o

puN ef3uga too

jT[lEpqU
puu asoo'j

pegusp iiiq

PU  OS( t

I I   I
l-   I   ll

tOC0

1 001

CCZO _-
Co LO i

too-

I- 0 to

o 01
010

&16 I

to -

t001-
CQOO

co-

1 CO OD

. . .

toot

00 .t

sasma

jo Joqu1nu-jugaeJd 0 01    C2,

UnI 10a ?odoaq

G;

4 4

0.4

+ iuauosTa+

+UO IWUI J    ?o

g10ggoA POOla to

poaa! Xsuoo e

s1eggA P0O?fi c?

IWmUou  o
seAsseA POOlif CO

peziull1q

puw asoo I I

imiqU t:, It
putt eguerq 8 t

',ellGljqU t

pug ago0l00 oo

T)eziullegq vo?
puw eguea -o

tot

scq N

0~ 01 to

Cq .

r-40

c? c
ro o to
0101

tot' t

0 tot

tOl
toto
0-0'
01to

tot- ?N

0 4)

to c0to   t- oo

6  q q    8- "

.   .  .   .   .

6m         U 8 8

44
A.s      E. E  E.

IC

.

-1

681

&6
CR
0

.pt

.P

00

00

4-4
Go b

0
00!;
0)

0t

* !;

00

-

0p
*0)

Drt

A

4sX

Fd
F-

. .

P. N. WAHI, U. K. LUTHRA AND V. L. KAPUR

+ JuGuISIl< I I I
+ Uo1WuU1UUI Nl

SIaSSU)A pooia o0 o i

Pa%Nluo!P ll: o C

SI0SOA POO?JI co C

r"IO   C4CC

I9411uuou m

SI9SSaA pOOI?  * ? ko

PGZU!IU)lAq 01 *

puu asooa ooc I

IBtU1lq cUr
pu- a     ooi I I
pug gsuo([   C

jo iaqtunu-juasajd C

iunlioo jodo(a

+ qtUsiIa -O.
+ uoflUtuIU1UIguj -

P04VIlp to7I?C

SIaSS@A poolE  Oka

plalsusou .

SIU)SSGA POOlife ko I

slassas P??lK- - ?

peziu;OA AII 0   to t
pozlui[iRAq  o e
pug i800@too

jvjjI!qU t
Pug auq( ,

i,lVllqU co ?

put; osuoa e H I

pUg "l0rq[ 000

p0z1lsU0q 00  I? I
pug' osuaq x

oeZIUIi?E 0'c

oi co r-i

OD
00

CO
0W
14t

4-

0 C
0v

00'l
0)o
00

.-0

o 0

00)

t)

I.

EH

5

-4

0

k;

AE

+ 1uouasta I
+ uoj!ummt;ijui

polipllu C?

SIOSSOA Pploa "

SIOSSOA P00111 a

put asoo'j N

ohqliqU '

pug aoueaq Co

co

pu; asooo'I

put; osutaC (

011
to

04

c-i
11

0I

1
ell

0
to

sasu)

jo joqtunu--uasoJd c

tunpoo liodaq

UL)
. 0

44

Cs
x6i
5

+ luuemaii

+uoi t;mmt;IuI

9pSSA posoja

It;ULA OOIU 0?
I9~SSQA POOIJi C*

peziu.Iuflt;X

put; iioolI

put; esoo'j
Paut osuoa E

to
0

r4

Co

to
0

co

Co0

to

Co
to11

0

Q;
C)
4S-0

.- C0 =n

682

1;
:0
4
a

Ct

Co

'lb

00t-

0G

00d

I.c%

01)
01

GO

U)

44"

0

SUBMUCOUS FIBROSIS OF THE ORAL CAVITY

0 0

lOQ0t-. 0

o  .   .   .

0D '00)

+     D

+    N

+  { Z

I  Io P- _   .

t o

0  .   .   .

+g   N

I t  s

.4 1

0

0

b

04

0   K

6hI   I

gJ 2

IP ,

O   1

2  I

*q * -

c4 4 Cs
C9 P- -

I0, .

I  I   o
+^

C)

0 O

. eb  .+

F6I

0

i?iI  {I:   - -

c -.-I

-  C4~~~~~ODC
o    0 o

COC) C)

ZM HE-E-'

0* * 00

,O cq P-

0 0co Co eq

04

0     "
a4 H H

683

0

. '

FP

0

0

CO
60

* a,

C.

e.)

.e *lea

EN

EH

P. N. WAHI, U. K. LUTHRA AND V. L. KAPUR

defect of connective tissue maturation whereas this was more often observed in
tobacco users.

Similar histologic and histochemical observations were seen in the deeper
corium although of less severity.

COMMENTS

In most of our cases the epithelium showed hyperplasia and less frequently
atrophy. Sirsat and Khanolkar (1957, 1962) also described a thickened epi-
thelium with deep invagination into subjacent lamina propria. Sharan (1959)
described hypertrophy with occasional areas of atrophy. In a large percentage
of our cases (69-2 per cent) of clinical group III, the epithelium showed atypical
epithelial hyperplasia (Table I). The epithelium in all the 4 cases of submucous
fibrosis associated with epidermoid carcinoma showed atypical epithelial hyper-
plasia. Cellular atypism has been described in the epithelium by Desa (1957),
although this was not correlated with the severity or extent of the lesion.

Pindborg et al. (1964) on the other hand, described atrophy of the epithelium
as the predominant feature. Although epithelial atrophy was an infrequent
finding in our cases, it is felt that it may follow hyperplasia as in skin carcino-
genesis where proliferative epithelial changes preceed atrophy (Stewart, 1959).

In our cases using tobacco smoking, chewing or both habits in this order, there
was a greater frequency of atypical epithelium compared to the non-tobacco users
(Table II). Tobacco tar paintings on the skin have been described to induce
progressive epithelial hyperplasia followed by areas of cellular atypism (Reddy,
Reddy and Rao, 1960). This observation helps in the understanding of the high
frequency of atypical epithelial hyperplasia in cases with the tobacco habit as
compared to non-tobacco users.

The more advanced clinical cases had an increased frequency and severity of
epithelial hyperplasia and atypism. V7arious views have been put forward as to
the development of epithelial changes. The alterations in connective tissue are
described to preceed the development of epitheliomas in sailors and ranchers
exposed to intense sunlight excessively (Stewart, 1959). It has been suggested
that stromal changes occur in preneoplastic sites that affect the epithelium by
interference with metabolic exchange, so that the epithelium remains viable but
deprived of essential organisational control (Orr, 1963). Further, altered connec-
tive tissue is thought to be responsible for epithelial hyperplasia and invasiveness
by liberation of hyaluronic acid from degenerate connective tissue or ground
substance (Gillman and Roux, 1955). Varoni (1951) postulated that the products
of degeneration have growth promoting properties.

Interesting observations were discerned when the type of keratinizing meta-
plasia was correlated with the site of the lesion and the habit of the patients.
Lesions involving the palate predominantly showed orthokeratosis, and those of
buccal mucosa parakeratosis. The frequent clinical recognition of the lesions
involving the palate (Wahi et al. 1966a, unpublished) may be facilitated to some
extent by the nature of keratinizing metaplasia, i.e. orthokeratosis.

A purely orthokeratotic type of epithelium was predominantly seen in non-
tobacco users in contrast to purely parakeratotic type of epithelium in tobacco
users. A parakeratotic type of epithelium was seen in 3 of our cases of submucous
fibrosis associated with carcinoma and a combination of para and orthokeratotic

684

SUBMUCOUS FIBROSIS OF THE ORAL CAVITY

types of epithelium in the fourth case. The type of keratinizing metaplasia in
relation to the habit has not been reported by any worker in cases of submucous
fibrosis. Pindborg et al. (1965) describe keratinization in less than 50 per cent of
cases of submucous fibrosis, with a high frequency of orthokeratotic and hyper-
orthokeratotic type of change.

A high mitotic count in parakeratotic epithelium as compared to orthokeratotic
epithelium and also an association of some parakeratotic leukoplakia with epithelial
atypia, carcinoma in situ or invasive carcinoma has been reported by Renstrup
(1963). Pindborg and Renstrup (1963) have describedlhyperplasiaandparakera-
tosis of epithelium in snuff induced leukoplakia. Acanthosis and hyperpara-
keratosis of palatal epithelium have been described in pipe smokers by Chapman
and Redish (1960). It is interesting to observe that the parakeratotic or hyper-
parakeratotic epithelium has been correlated with tobacco use in one way or
another. Further, the association or co-existence of this type of epithelium to
atypia and neoplasia is more frequently recognized.

The stratum granulosum was more frequently patchy or absent in tobacco
users than in non-tobacco users (Table III). The stratum granulosum was seen
in areas of orthokeratotic epithelium, while it was absent or indistinct in the
parakeratotic type of epithelium. Spongiosis of the stratum malpighi was
frequently observed in tobacco smokers while it was a rare feature in non-tobacco
users (Table III).

The rete pegs were more frequently pointed as one considered cases from group
I to group III. Moreover, they were more frequently pointed in tobacco users
(Table III). Deep invagination of epithelium into subjacent lamina propria in
cases of submucous fibrosis has been described by Sirsat and Khanolkar (1962).
Liquefaction in basal cell layer was not observed in our cases as reported by
Sharan (1959) and Pindborg et al. (1965).

Basement membrane was distinctly discernible in a majority of the group I
and group II cases, and focally indistinct in a fair number (46 1 per cent) of group
III cases (Table I). Interestingly, the basement membrane was distinctly discern-
ible in all non-tobacco users whereas, it was indistinct in 12 cases, i.e. 15-8 per cent
of tobacco users. Loss of sharp definition of the basement membrane has been
described in leukoplakia of the palate in women smoking chutta (Reddy and Rao,
1957). Stewart (1959) described the ability of proliferating epithelial cells during
skin carcinogenesis to dissolve the basement membrane.

It has been observed in recent studies (Ashworth, Stembridge and Leibel, 1961)
on the basement membrane of cervix in intraepithelial and invasive carcinoma
that the basement membrane visualized by electron microscopy is a true membrane
and the cytoplasmic penetration during invasion occurs only at the sites where the
basement membrane is defective. It has been suggested that malignant cells in
some way mechanically disrupt or destroy the membrane or they produce a
metabolite which may depolymerize or otherwise destroy the basement membrane,
or the connective tissue may fail in the perpetual reconstruction of basement
membrane.

The number of mitotic figures in the epithelium increased from cases of clinical
group I to clinical group III and reached a maximum in 4 cases of submucous
fibrosis whiclh were associated with epidermoid carcinoma, i.e. 6 to 13 per 10 high
powNer fields. Considering the number of mitotic figures according to the habit
and the type of keratinization. these were more frequent in tobacco users who

30

685

P. N. WAHI, U. K. LUTHRA AND V. L. KAPUR

predominantly showed a parakeratotic epithelium in contrast to non-tobacco
users who frequently revealed an orthokeratotic type of epithelium. High
mitotic activity in parakeratotic epithelium as compared to orthokeratotic type
of epithelium has been reported in cases of leukoplakia (Renstrup, 1963). Reddy
et al. (1960) reported definite evidence of increased mitotic and amitotic division
in interscapular skin of mice treated with tobacco tar and heat, suggesting the
influence of these two factors in causing increased and rapid epithelial proliferation.

Striking alterations were discerned in the juxta-epithelial tissue and less so
in the deeper corium. In most of the cases of submucous fibrosis, the juxta-
epithelial tissue showed dense hylanization and fibrosis and only infrequently a
loose fibrillar type of subepithelial tissue.

Connective tissue changes in cases of submucous fibrosis have been variably
interpreted. Sirsat and Khanolkar (1957 and 1962), Sharan (1959), and Rao
(1962) have described marked increase in dense collagen in the sub-epithelial
tissue. Sirsat and Khanolkar (1957 and 1962) and Sharan (1959) have also
described hyalinization of the connective tissue. The changes in connective
tissue have been interpreted as fibrinoid degeneration by Sharan (1959) and elastotic
degeneration by Sirsat and Khanolkar (1962). Rao (1962), on the other hand,
did not find elastotic or fibrinoid degeneration.

A distinct increase in the frequency of dense hyalinized juxta-epithelial tissue
was observed in cases from group I to group III. The majority of the patients
with the habit of tobacco chewing and smoking and of tobacco chewing alone
showed dense and hyalinized type of juxta-epithelial tissue.

In the majority of cases of submucous fibrosis, the blood vessels in the juxta-
epithelial connective tissue were constricted. Interestingly, the number of
constricted blood vessels in the juxta-epithelial connective tissue was more in
group III cases than in group I cases. This feature was also more common in
tobacco users as compared to non-tobacco users.

The majority of the cases of clinical group III and those with the habit of
tobacco chewing and smoking gave a markedly positive metachromasia with
alcoholic toluidine blue indicative of acid mucopolysaccharides (Pearse, 1960).
An increase in the mucopolysaccharide content of connective tissue in general
has been reported in guinea-pigs after inhalation of tobacco smoke (Lupu and
Velikan, 1962). Accumulation of mucopolysaccharides in the dermis in carcinogen
treated rabbits has been described by Maltoni and Prodi (1960).

It is suggested that the connective tissue changes may preceed any epithelial
anomaly or may be concomitant. The epithelial and connective tissue changes
seem to depend on the combined effect of tobacco on tissues preconditioned by
vitamin deficiencies (Wahi et al., 1966b, unpublished). However, the epithelial
changes may be aggravated by the abnormalities of underlying connective tissue
and blood vessels, which may act by interference with metabolic exchange or the
direct effect of the products of degeneration or altered metabolism. The products
of degeneration have been considered to have growth promoting properties
(Varoni, 1951), and thus the epithelial changes may be secondary to the connective
tissue changes.

SUMMARY

A detailed histomorphological study in 104 cases of submucous fibrosis is
described. These observations are presented with special reference to the severity

686

SUBMUCOUS FIBROSIS OF THE ORAL CAVITY        687

and extent of the lesion and the habits (tobacco smoking, tobacco and betal nut
chewing) of the patients. A close correlation was found between the clinical
severity of the cases and the histopathological changes in the biopsy material.
Patients using tobacco in one form or the other i.e. smoking, chewing or both,
showed more marked histopathological changes. Four cases of submucous
fibrosis were associated with epidermoid carcinoma.

REFERENCES

ASHWORTH, C. T., STEMBRIDGE, V. A. AND LEIBEL, F. N.-(1961) Acta cytol., 5, 369.
CHAPMAN, I. AND REDISH, C. H.-(1960) A.M.A. Archs Path., 70, 133.
DESA, J. V.-(1957) Ann. Otol. Rhinol. Lar., 66, 1143.

GILLMAN, T., PENN, J., BRONKS, D. AND Roux, M.-(1954) Nature, Lond., 174, 789.
GILLMAN, T,. AND Roux, M.-(1955) A.M.A. Archs Path., 59, 733.
LuPu, N. G. AND VELIKAN, K.-(1962) Arkh. Patot., 24, 2.

MALTONI, C. AND PRODI, G.-(1960) in ' Recent contribution to Cancer research in

Italy " edited by Bucalossi, P. and Veronesi, U., Milan (Casa editrice ambosina)
Vol. 1, p. 49.

ORR, J. W.-(1963) in ' Conference on biology of cutaneous cancer ' Natn. Cancer Inst.

Monogr. No. 10, edited by Urbach, F., Bethesda, 14, Maryland (U.S. Department
of Health, Educational and Welfare) p. 531.

PEARSE, A. G. E.-(1960) 'Histochemistry-Theoretical and applied', 2nd. edition,

London (Churchill).

PINDBORG, J. J., CHAWLA, T. N., SHRIVASTAVA, A. N. AND DAYA GUPTA-(1964) Acta

odont. scand., 22, 679.-(1965) Acta odont. scand., 23, 277.

PINDBORG, J. J. AND RENSTRuP, G.-(1963) Acta derm.-vener, 43, 271.
RAO, A. B. M.-(1962) Br. J. Surg., 50, 23.

REDDY, D. G. AND RAO, V. K.-(1957) Indian J. med. Sci., 11, 791.

REDDY, D. G., REDDY, D. B. AND RAO, P. R.-(1960) Cancer, N.Y., 13, 263.
RENSTRuP, G.-(1963) Acta odont. scand., 4, 333.
SHARAN, J.-(1959) Indian J. Path. Bact., 2, 150.

SIRSAT, S. M. AND KHANOLKAR, V. R.-(1957) J. Path. Bact., 73, 439.-(1960) J. Path.

Bact., 79, 53.-(1962) Indian J. med. Sci., 16, 189.

STEWART, H. L.-(1959) in 'The physiopathology of Cancer', 2nd edition, edited by

Homberger, F., New York (Hoeber & Harper) p. 3.
VARONI, C. L.-(1951) Scientia med. ital., 2, 273.

WEINMANN, J. P. AND MEYER, J.-(1959) J. invest. Derm., 32, 87.

				


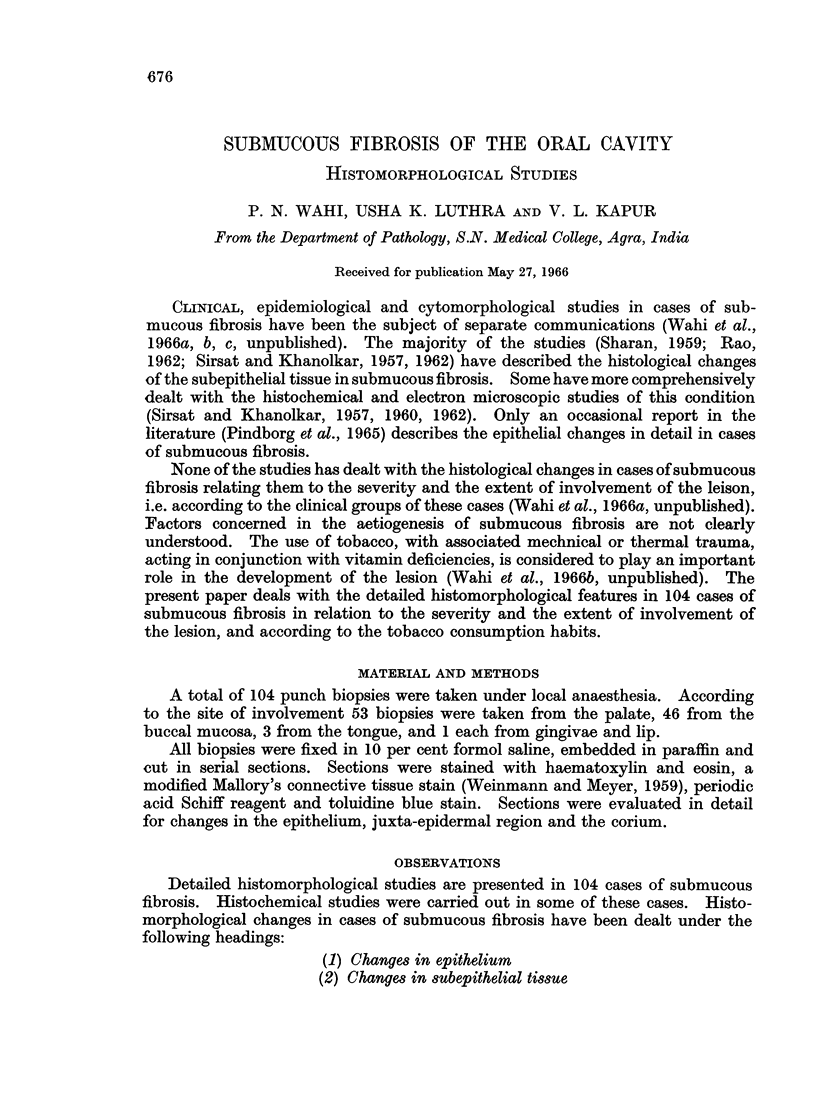

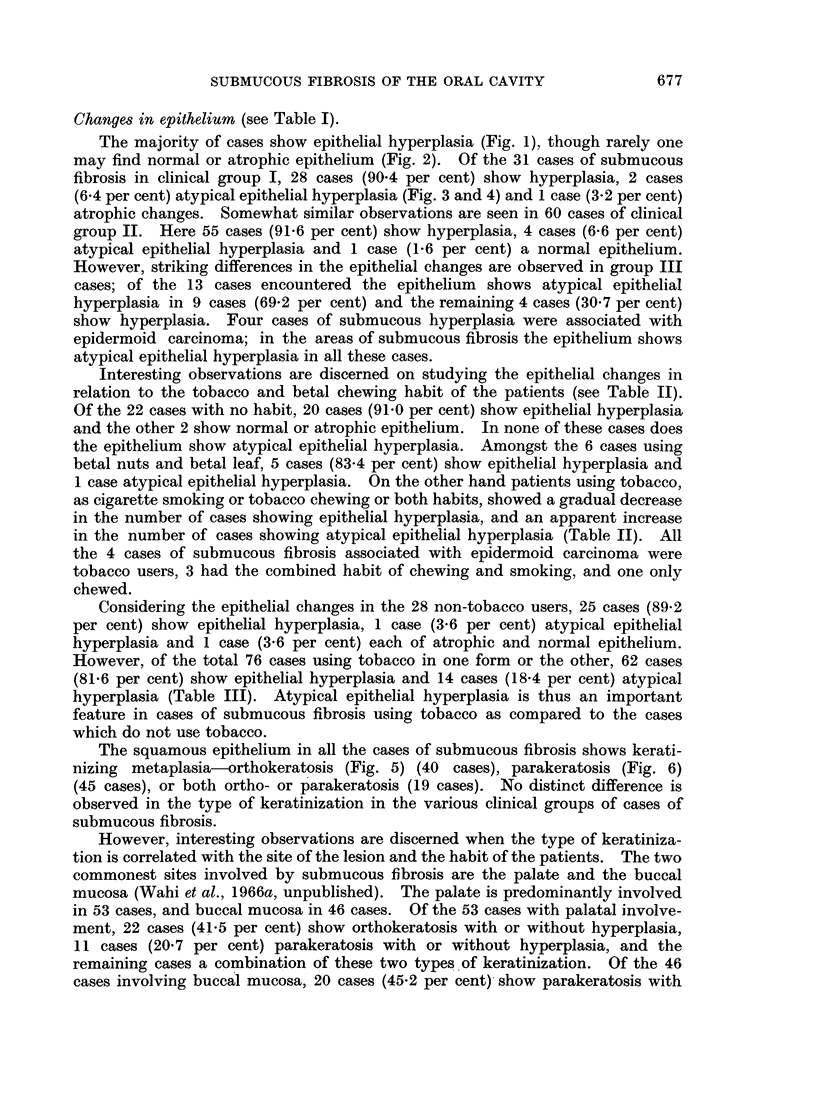

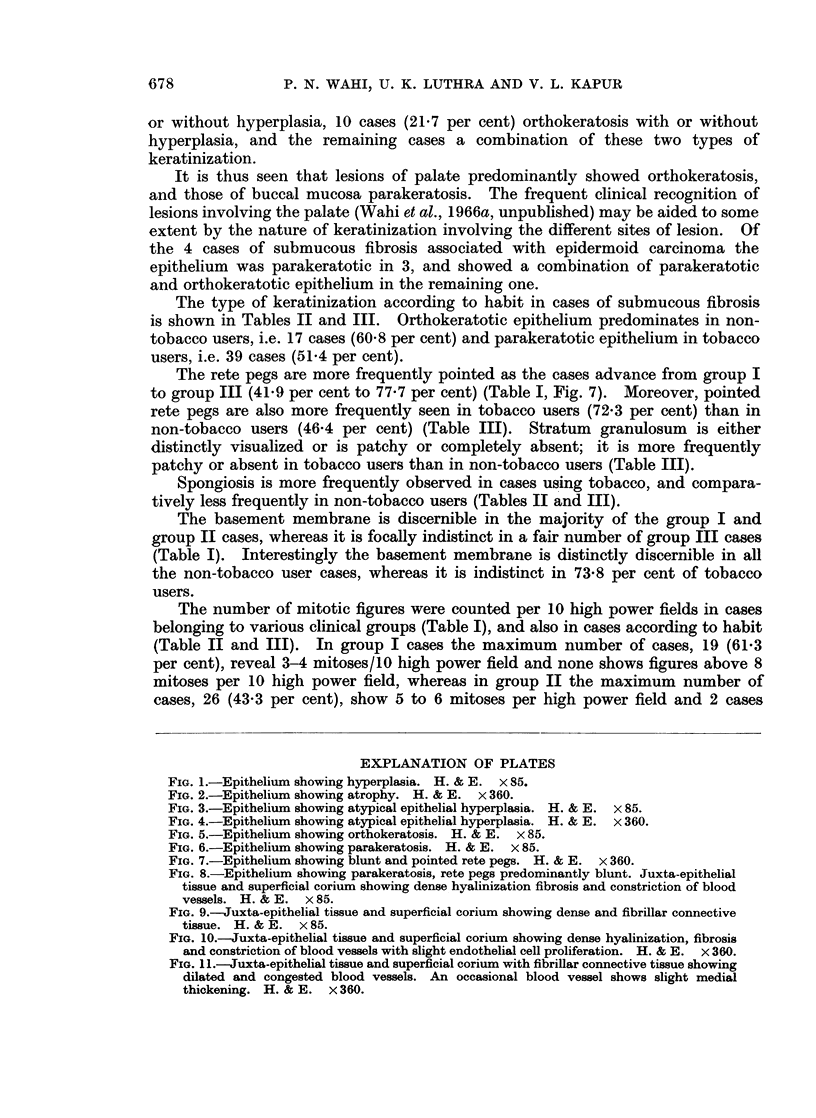

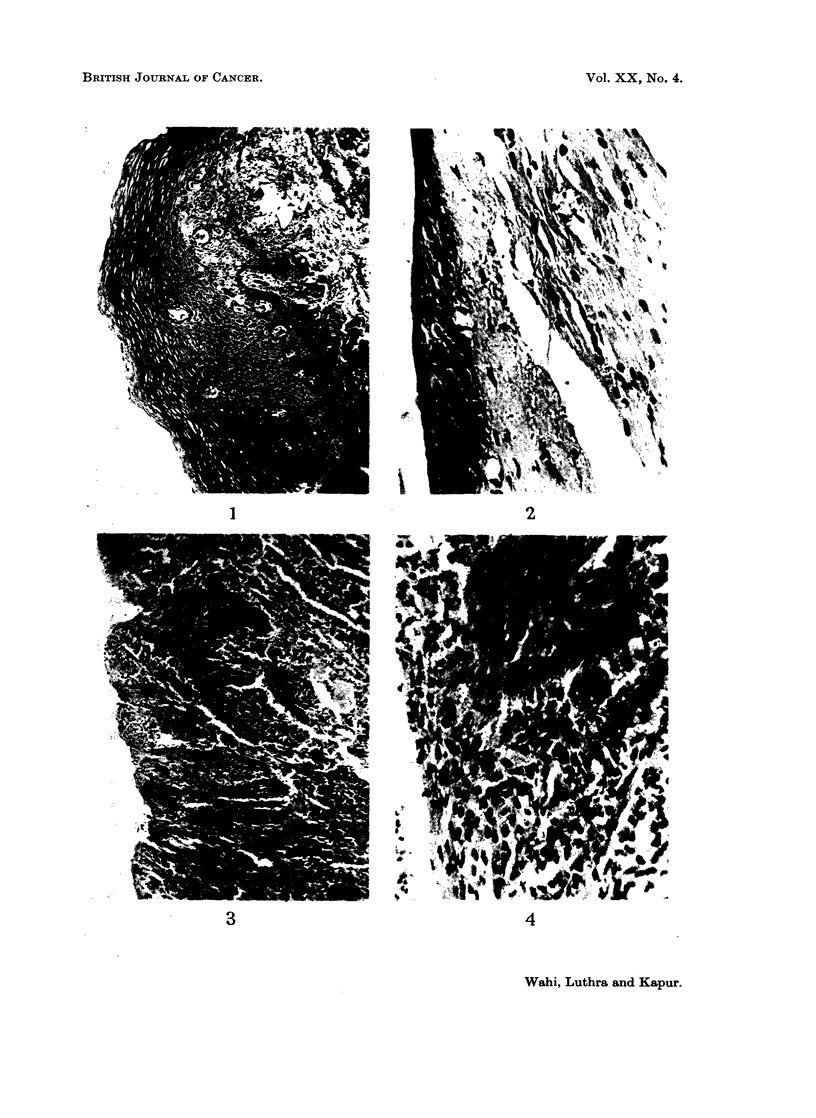

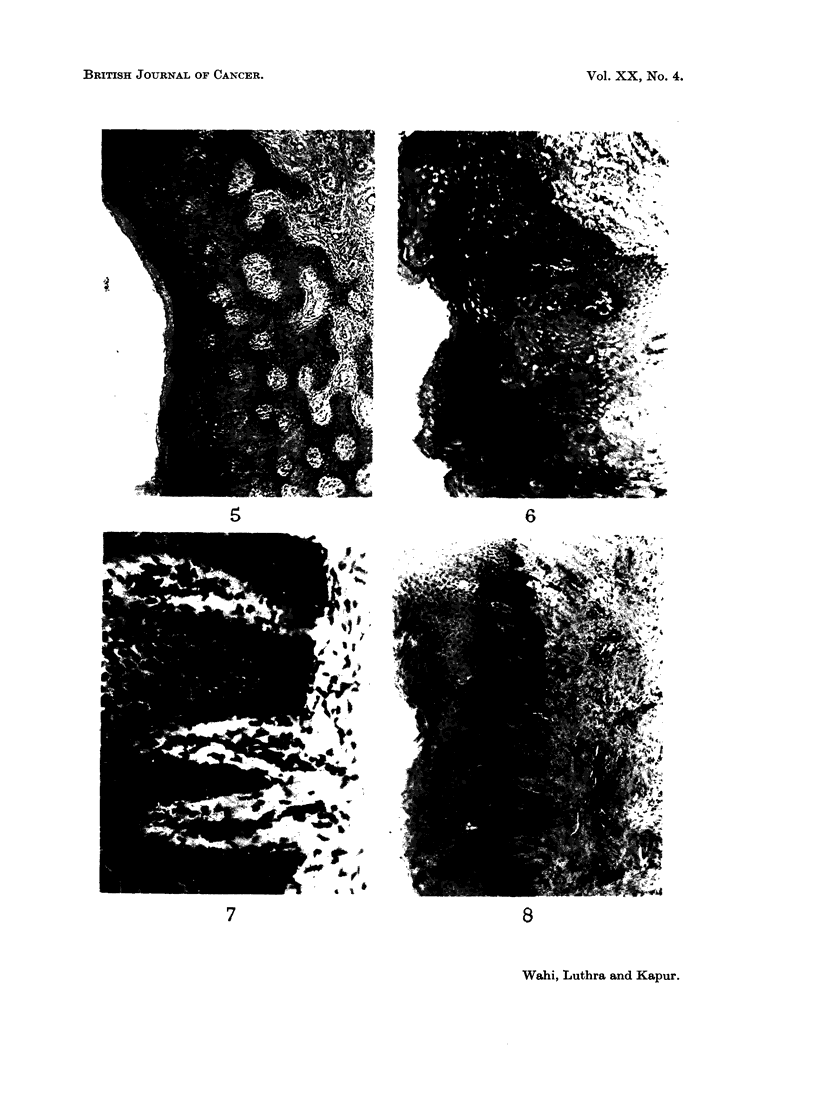

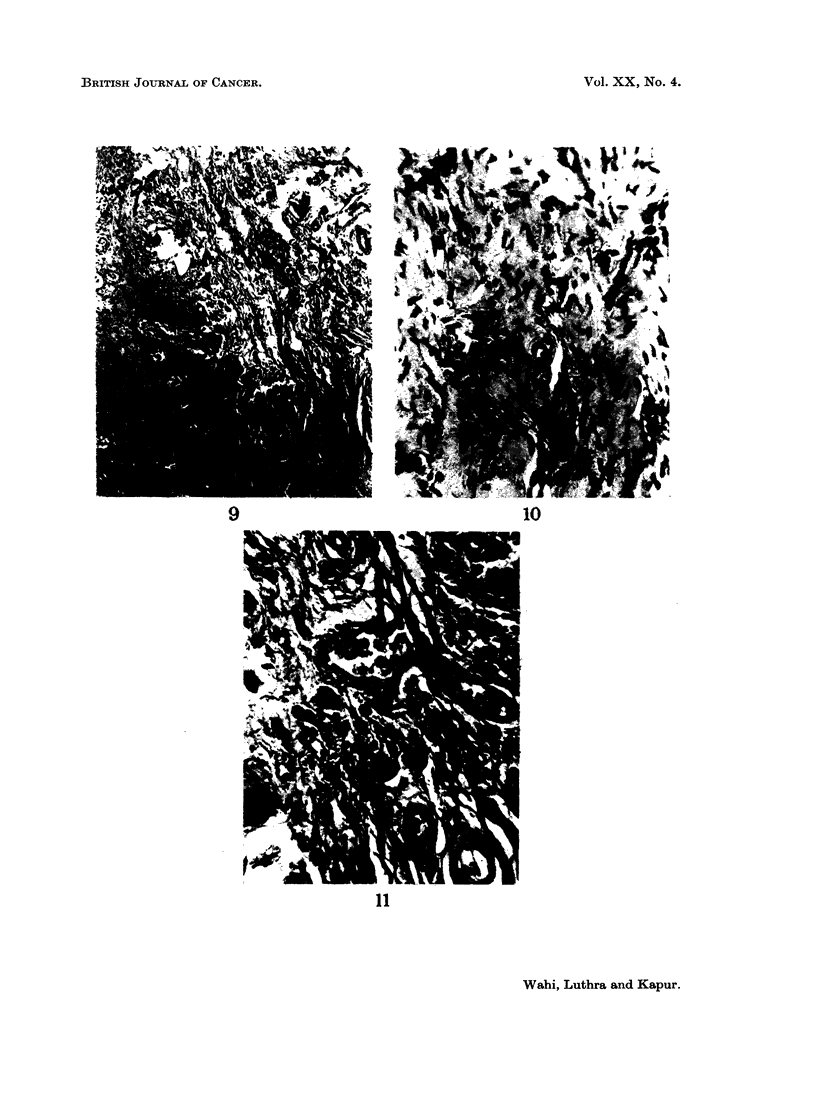

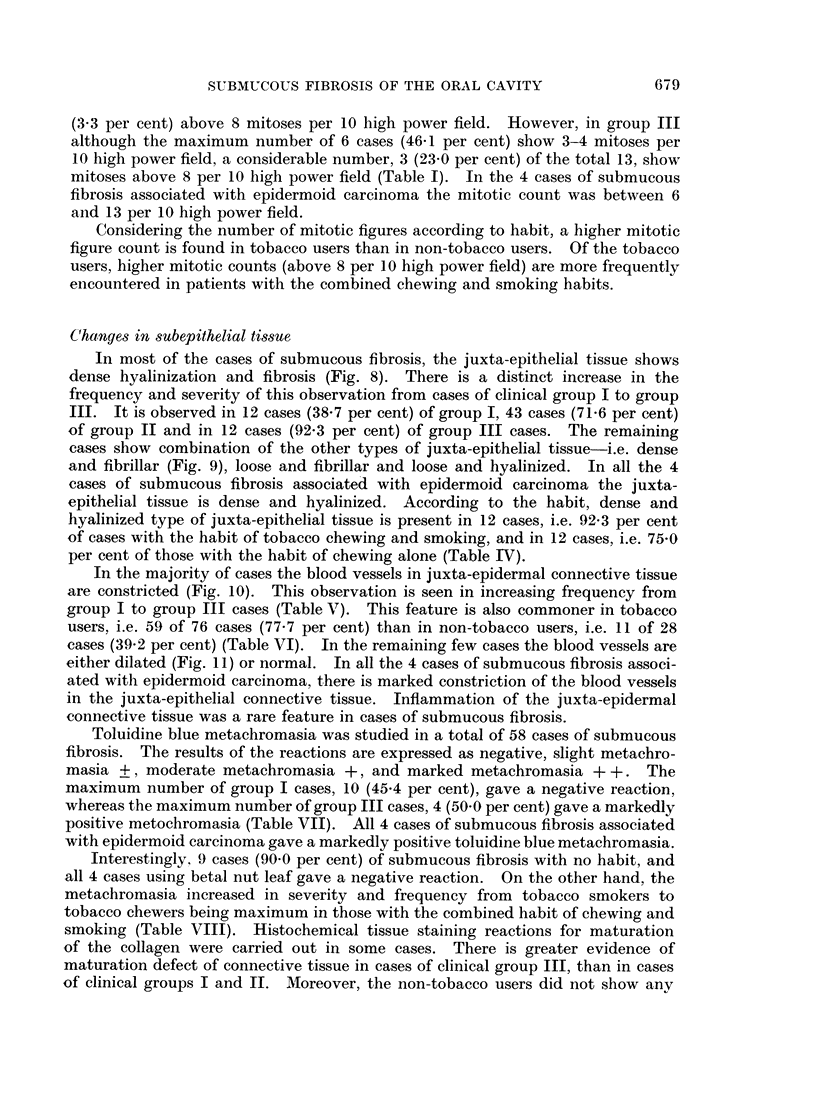

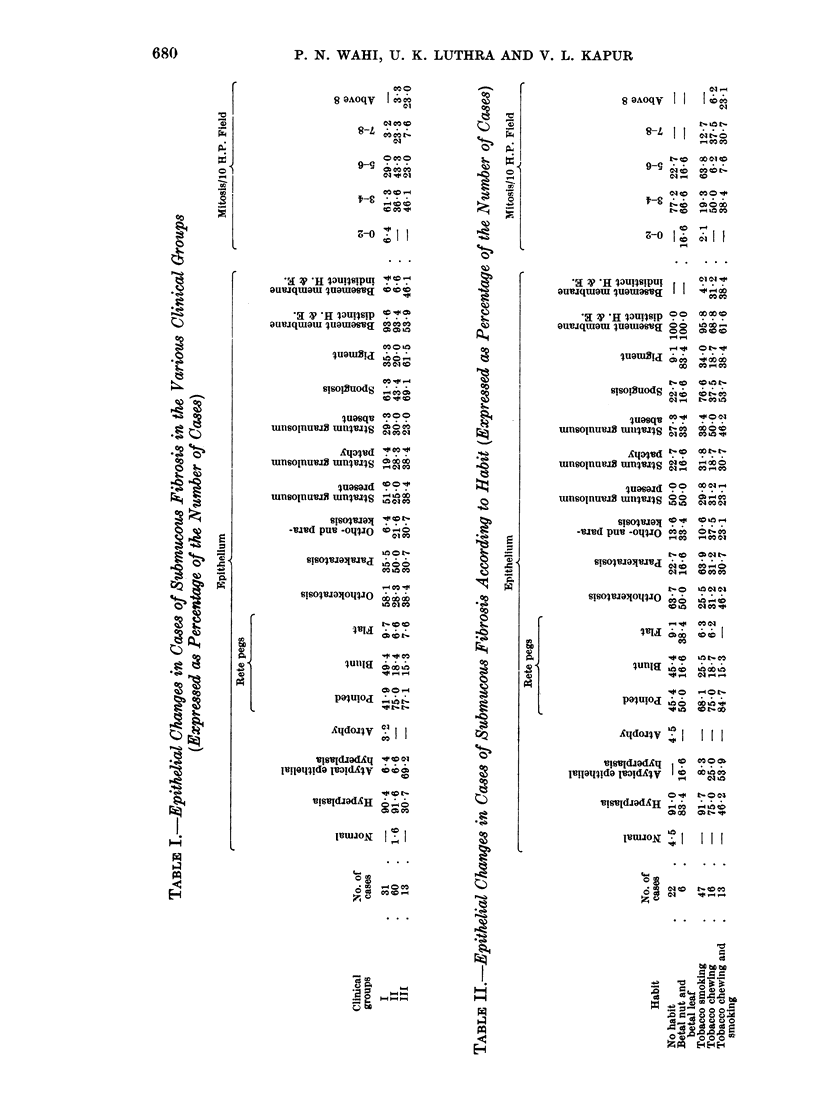

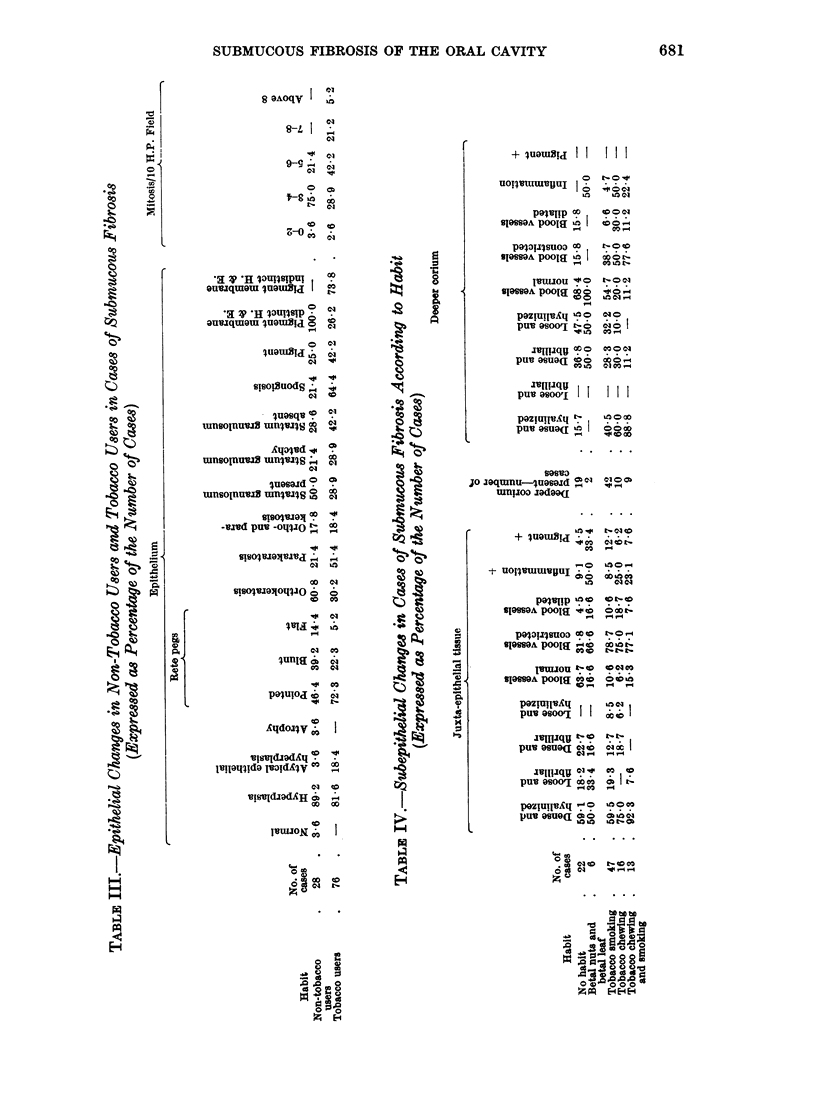

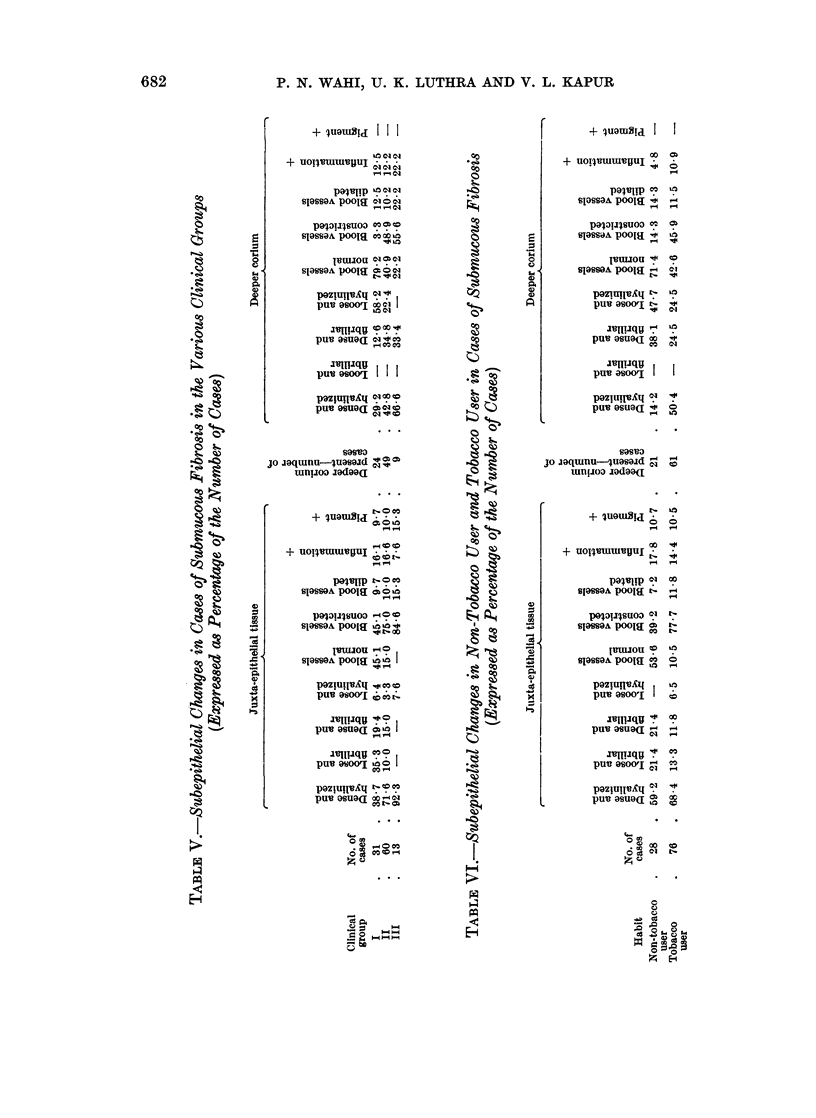

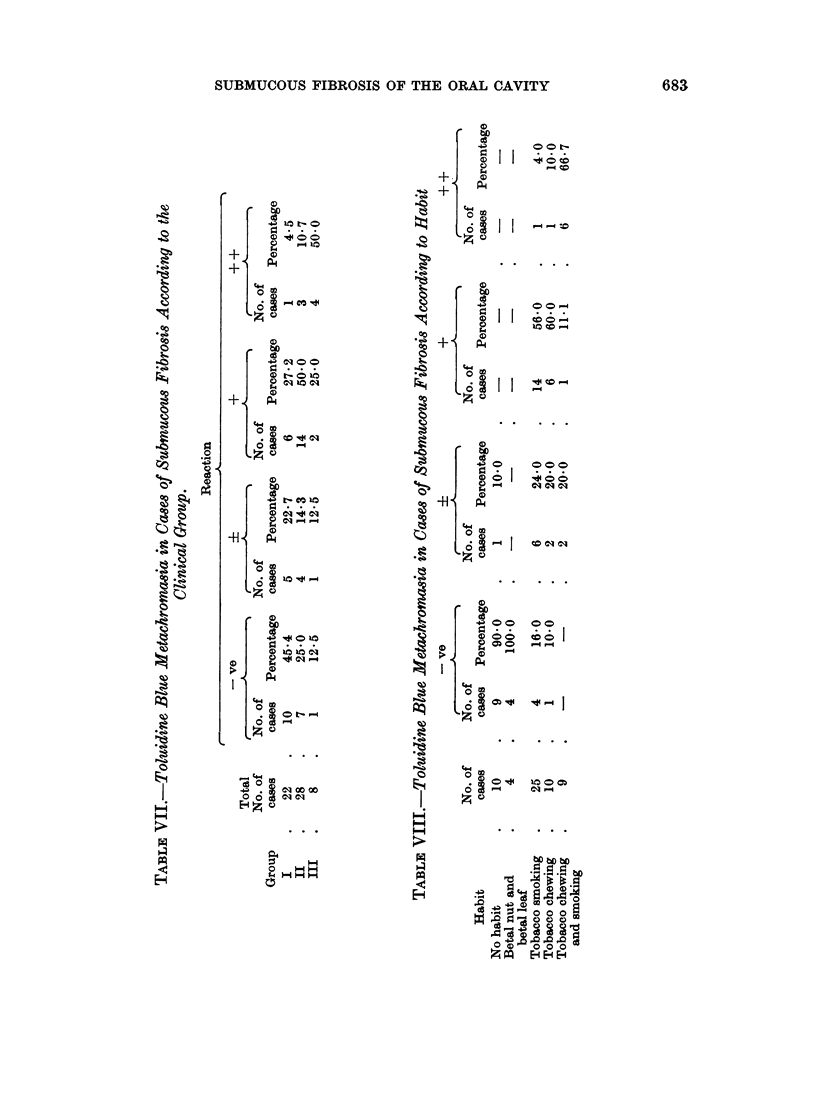

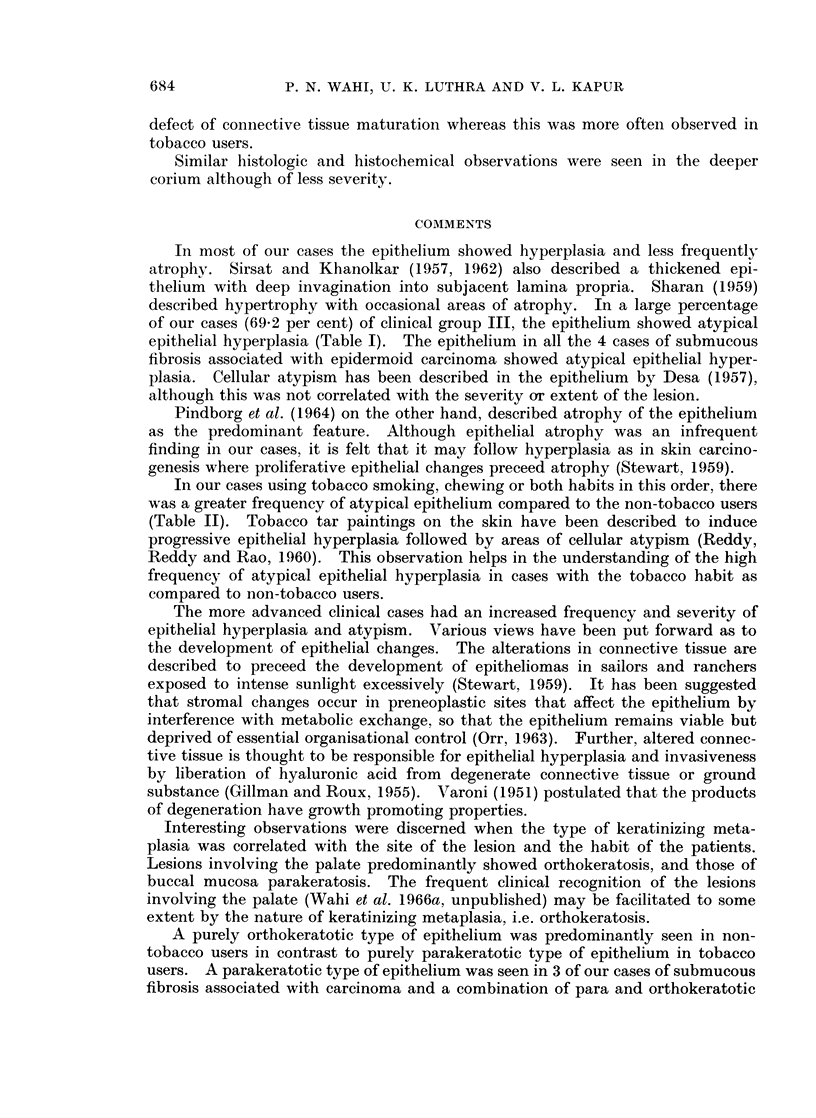

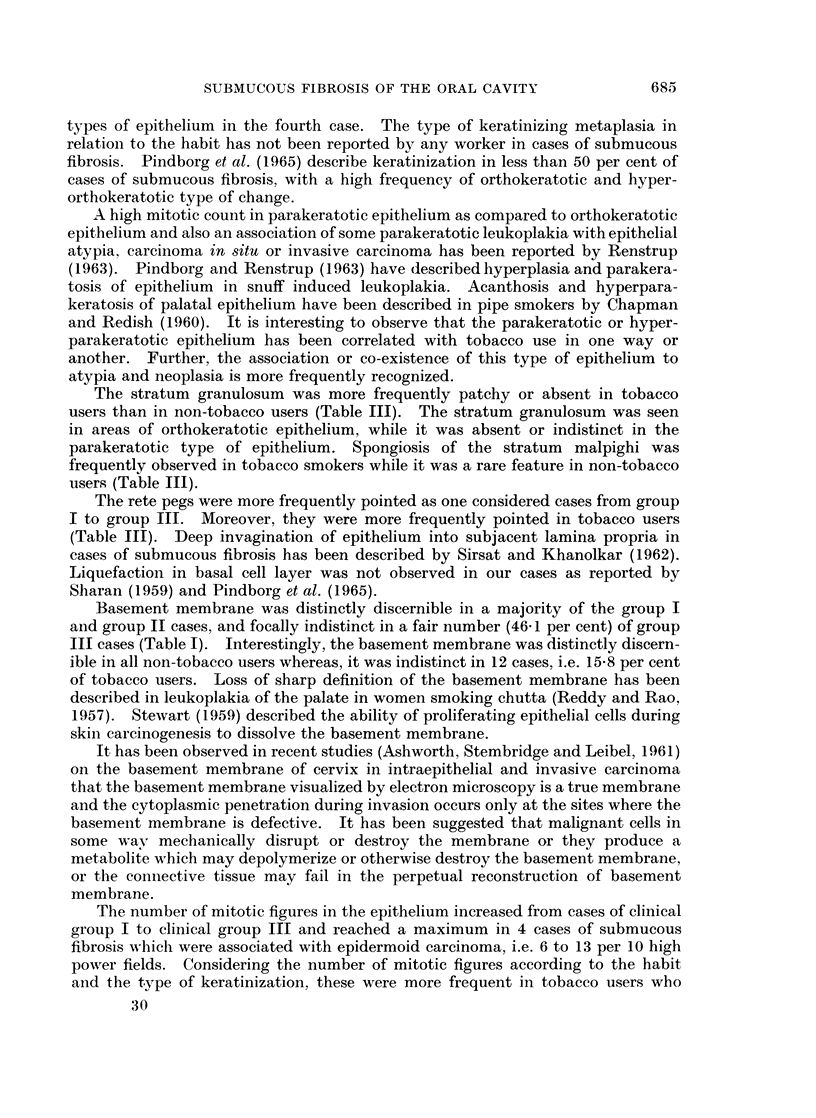

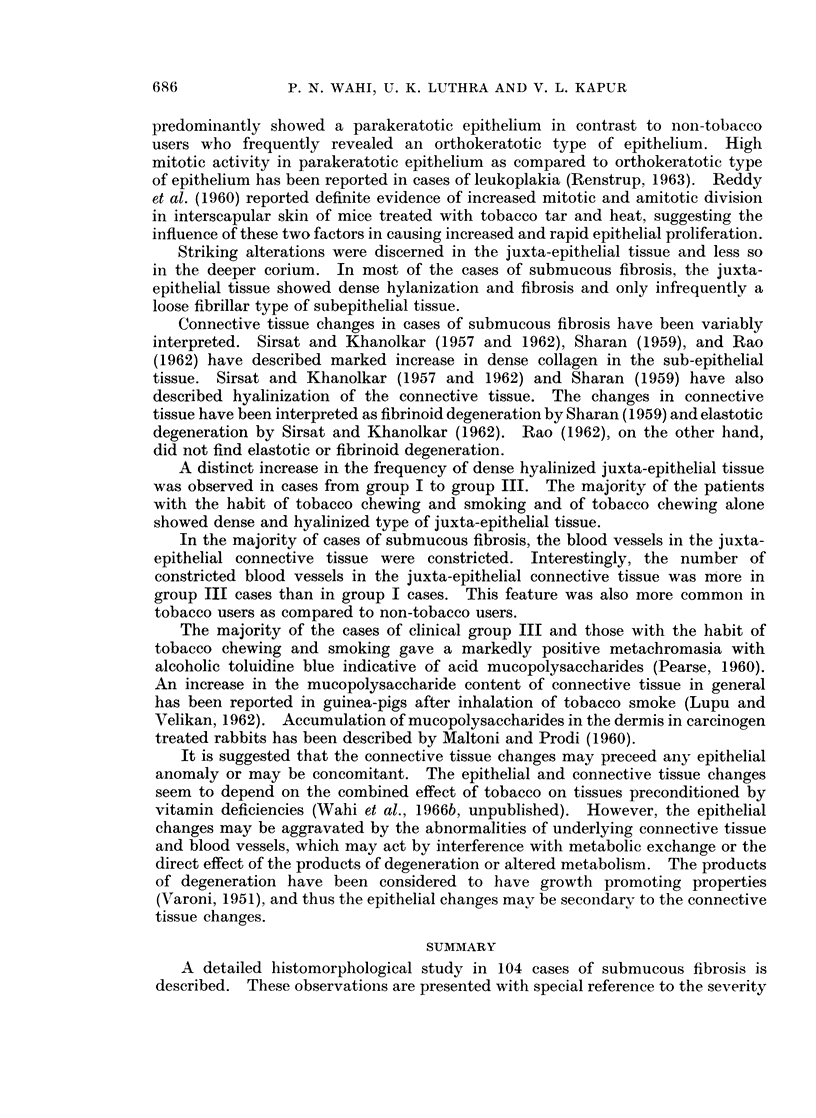

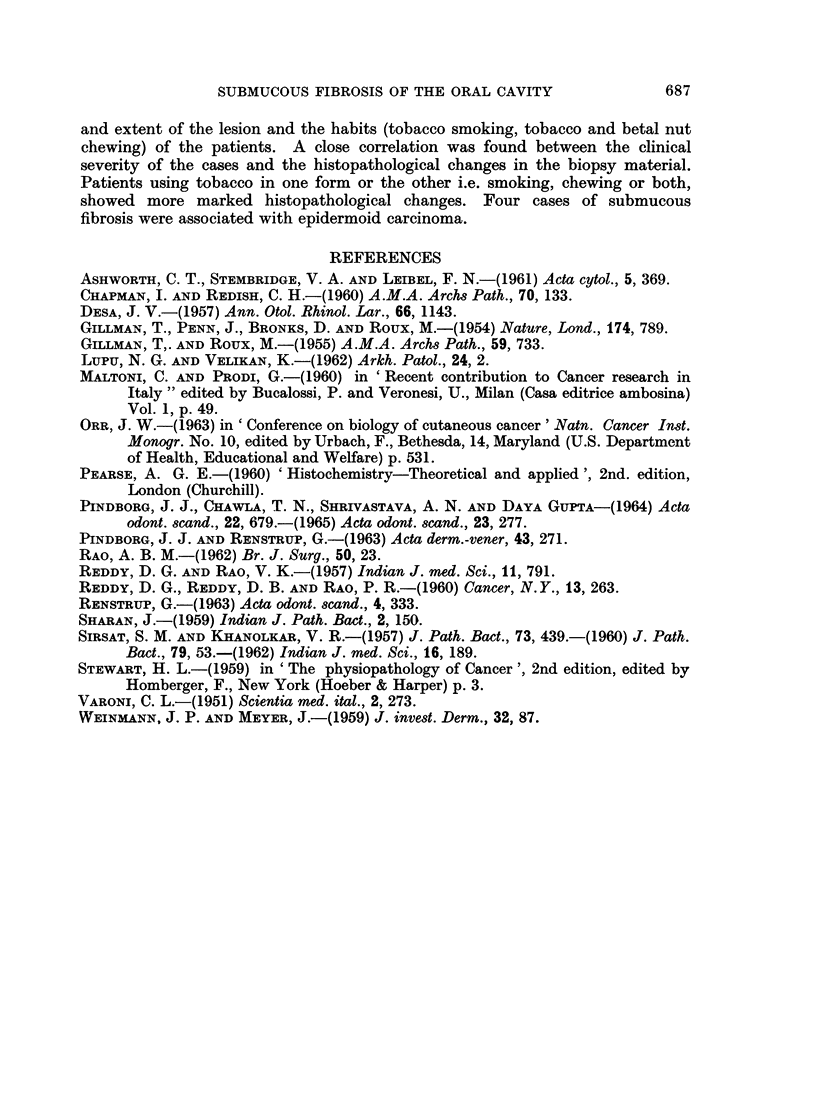

